# Improving Clozapine Blood Monitoring in Inpatient Psychiatry: A Targeted Quality Improvement Initiative

**DOI:** 10.1192/bjo.2025.10429

**Published:** 2025-06-20

**Authors:** Saroop Raja, Ammar Lakda

**Affiliations:** 1 Birmingham and Solihull Mental Health NHS Foundation Trust, Birmingham, United Kingdom; 2 Department of Anesthesiology Research, Cleveland Clinic, Cleveland, Ohio, USA; 3 The Outcomes Research Consortium, Houston, Texas, USA

## Abstract

**Aims:** Clozapine is critical for treatment-resistant schizophrenia, yet inappropriate monitoring can have severe physical and mental health consequences. Toxicity can result in bowel obstruction, myocarditis, and life-threatening agranulocytosis, while subtherapeutic levels increase the risk of relapse. Ensuring appropriate blood monitoring is a national concern in inpatient psychiatric care.

In response to missed full blood counts (FBCs) and inappropriately ordered clozapine levels in our trust, this quality improvement project (QIP) aimed to improve adherence to Trust Guidelines. The goal was to ensure that FBCs were sent routinely while clozapine levels were only requested upon consultant guidance, reducing inappropriate tests from 53% at baseline to zero by April 2024. The intervention was targeted towards nurses responsible for ordering routine blood tests.

**Methods:** An initial audit of all clozapine-related blood tests was conducted between 1 November 2023 and 1 January 2024. The intervention was implemented in January 2024 which included a 20-minute training session on clozapine blood monitoring, delivered to nurses across three separate days to maximize attendance. The training covered clozapine pharmacology, monitoring requirements, appropriate indications for clozapine level testing, and practical steps for arranging blood tests. Additionally, an aide-memoire was placed at key ward locations, including near the laptop used for blood test requests and in the nursing station. The primary outcome was the number of inappropriate clozapine levels sent over a three-month period, while the secondary outcome assessed staff confidence in clozapine blood monitoring through pre- and post-training questionnaires.

**Results:** The project successfully achieved its primary aim. During the re-audit period, FBCs were sent routinely for all patients on clozapine. Clozapine levels were only sent upon consultant guidance for suspected toxicity, with no inappropriate requests. Regarding the secondary outcome, staff reported increased confidence and understanding of clozapine monitoring protocols based on questionnaire responses.

**Conclusion:** Engaging frontline staff in understanding the rationale behind clinical investigations was key to achieving sustained practice change. Providing structured, accessible training ensured that staff directly responsible for ordering routine blood tests felt confident in making appropriate testing decisions. This highlights the broader importance of equipping frontline allied healthcare workers with both the knowledge and the reasoning behind clinical guidelines to optimize patient safety and care. To maintain long-term improvements, periodic training refreshers and integrating these principles into standard staff education programmes are recommended. Future initiatives should explore similar training approaches across other clinical settings where test ordering decisions impact patient outcomes.

